# Application and mechanism of anti-VEGF drugs in age-related macular degeneration

**DOI:** 10.3389/fbioe.2022.943915

**Published:** 2022-09-23

**Authors:** Dawei Song, Ping Liu, Kai Shang, YiBin Ma

**Affiliations:** Tai’an City Central Hospital, Taian, China

**Keywords:** mechanism, anti-VEGF drugs, age-related macularde, treatment, choroidal neovascularization

## Abstract

Age-related macular degeneration (AMD) is the leading cause of blindness in the elderly. The incidence rate increases with age in people over 50 years of age. With the advent of China’s aging society, the number of patients is increasing year by year. Although researchers have done a lot of basic research and clinical research on the pathogenesis and treatment of AMD in recent years, the pathogenesis of AMD is still controversialdue to the complexity of the disease itself. AMD is the primary cause of blindness in the elderly over 50 years old. It is characterized by the formation of choroidal neovascularization (CNV) and the over secretion of vascular endothelial growth factor (VEGF) as its main mechanism, which can eventually lead to vision loss or blindness. The occurrence and development of AMD is an extremely complex process, in which a large number of regulatory factors and cytokines are involved. Most of the existing treatments are for its concomitant CNV. Targeted VEGF drugs for neovascularization, such as Lucentis and Aflibercept, are the first-line drugs for AMD. Their application has greatly reduced the blinding rate of patients. However, there are still some patients who have no response to treatment or cannot maintain their vision after long-term treatment. Frequent injection also increases the risk of complications and economic burden. In order to further improve the quality of life and long-term prognosis of AMD patients, a variety of new treatmentshave been or will be applied in clinic, including combined treatment with the same or different targets to improve the curative effect, change or simplify the mode of medication, inhibit VEGF receptor tyrosine protein kinase and so on. This article provides a brief review of the research progress of anti-VEGF drugs and their mechanisms for the treatment of AMD, it is expected to provide a better treatment plan for AMD treatment.

## Introduction

Age-related macular degeneration (AMD) is a disease that causes progressive and irreversible loss of central vision and poses a serious threat to the vision of the elderly. There are more than 5 million AMD patients in China. With the improvement of my country’s economy, medical level and average life expectancy, the prevalence of AMD has been rising (Ambati, 2011; Vogl et al., 2021). According to the fundus manifestations, AMD is divided into exudative AMD and atrophic AMD. Exudative AMD accounts for about 10% of AMD, but the damage to vision is much greater than atrophic AMD (Zhang et al., 2003; Mettu et al., 2020a; Arjunan et al., 2021). Atrophic AMD is characterized by geographic atrophy in the macular region. According to the pathological changes, AMD can be divided into two categories: wet and dry. Wet AMD is mainly characterized by choroidal neovascularization (CNV) formation, retinal pigment epithelium (RPE) detachment, macular hemorrhage and edema, also known as exudative or neovascular AMD. Wet AMD accounts for only 10% of AMD, but the harm is much greater than dry AMD (Montorio et al., 2021). With the aging population intensifies, the harm of AMD will further increase. At present, the treatment of wet AMD mainly targets CNV, and the treatment methods are mainly drug treatment, photodynamic therapy (PDT) and surgery.

PDT is the main treatment before anti-VEGF drugs are used in wet AMD. It destroys CNV while preserving function of RPE cells and neural retina without causing central visual impairment and visual field defect (Stenirri et al., 2012; Wei and Li, 2020; Hu et al., 2021). However, PDT is often accompanied by complications such as necrosis and apoptosis of corneal cells, temporary visual impairment, photosensitivity reactions, and has disadvantages such as easy recurrence and high price. Surgical treatment is highly invasive and postoperative visual recovery is affected to some extent (Yildirim et al., 2004). Intravitreal injection of anti-VEGF is widely used as the first-line drug in clinicfor the treatment of AMD because of its low visual impairment and few adverse effects, but there are still problems such as the need for repeated multiple treatments, the lack of response to treatment in some patients, and the increased risk of repeated intravitreal injection (Gale et al., 2003; Tavakoli et al., 2020; Wei et al., 2020). The development of new drugs, improvements in dosage forms and medication methods are expected to improve these deficiencies, such as the application of eye drops, oral preparations and anti VEGF drug extended-release devices, which brings hope to reduce the use of anti-VEGF drugs (Zhang et al., 2014; ClearkinLRamasamy et al., 2019). This article provides a brief review of the research progress of anti-VEGF drugs and their mechanisms for the treatment of AMD.

## Characteristics of vascular endothelial growth factor

VEGF is a kind of homodimeric glycoprotein linked by disulfide bonds. It has heparin binding activity, which promotes the mitosis of vascular endothelial cells to promote angiogenesis. VEGF is widely distributed in the brain, kidney, liver, eye and other tissues and organs of the human body. The retinal pericytes, pigment epithelial cells, endothelial cells and ganglion cells in the eyes can express VEGF, which plays an important role in maintaining the integrity of ocular blood vessels, but overexpression will promote the abnormal proliferation of blood vessels (Weber et al., 1994; Ma et al., 2021). Its gene family members mainly include VEGF-A, VEGF-B, VEGF-C, VEGF-D, VEGF-E and placental growth factor. Among them, VEGF-A plays an important role in neovascularization and increasing vascular permeability (Bhisitkul, 2006). The intrinsic biological characteristics of VEGF include: 1) Promoting endothelial cell mitosis and angiogenesis. 2) Increasing the permeability of vascular endothelium, making plasma protein overflow out of blood vessels, causing fibrin coagulation, and forming a temporary matrix for angiogenesis. Meanwhile, promoting stromal cells to further form mature vascular matrix, so as to promote angiogenesis. Changing the extracellular matrix and indirectly promoting angiogenesis. 3) Having neuroprotective effectby inhibiting neuronal apoptosis, promoting the proliferation of nerve cells and protecting retinal nerve fibers. Having vascular protective effect by antithrombotic effect (DorY and Keshet, 2001).

## Pathogenesis

The risk factors for AMD are mainly age, race, smoking, drinking, sunlight exposure, inner eye surgery and genetic factors, etc. Most scholars believe that the joint action of environmental factors and genetic factors leads to the occurrence of AMD (Arjunan et al., 2021). The pathogenesis of AMD is complex and controversial. Now we mainly introduce the following pathogenesis.

### Aging and metabolic changes of retinal pigment epithelium

With the decline of systemic function in the elderly, RPE also changes. Mettu et al. (2020a) found that apoptosis of RPE cells in the macula region increases with age, and the activity of lysosomal system in RPE cells is positively correlated with age. Aging initiates the lysosomal system, which leads todamage of RPE cells. RPE cells in human eyes are able to provide metabolic support for retinal photoreceptor cells. The outer segments of retinal photoreceptor cells renew periodically, and the shed substances are swallowed and cleared by RPE cells. The apoptosis and damage of RPE cells reduce their “cleaning” ability. Metabolites accumulate in the inner layer of Bruch’s membrane, forming vitreousmembrane warts that damage the adjacent retinal tissue and lead to retinal tissue atrophy, forming a vicious cycle that eventually causes calcification and rupture of Bruch’s membrane, resulting in the formation of CNV (Montorio et al., 2021). The aging of RPE cells also breaks the balance of enzymes in its extracellular matrix, causing the local RPE extracellular matrix in the macular region to gather on the Bruch’s membrane, resulting in the thickening of Bruch’s membrane, reducing retinal blood supply, stimulating phagocytes to produce angiogenic factors, resulting in the formation of CNV, which is seriously harmful to vision.

### Light damage and oxidative damage

When looking at objects, the macula is always under light, so some scholars have proposed the mechanism of light damage (Wei and Li, 2020). Studies have shown that long-term irradiation of light will increase the concentration of free radicals in the eye. The original physiological balance is broken and the outer segments of photoreceptors are the first to be attacked by free radicals (Wei and Li, 2020). Light damage and oxidative damage cause retinal cell damage and apoptosis, which in turn lead to the occurrence and development of AMD. Research have (Hu et al., 2021) reported that hydrogen peroxide was used to induce the expression of recombinant ferritin, mitochondrial (FtMt) in acute retinal pigment epithelium (ARPE) cells, and then the overexpression of FtMt induced by oxidative stress was detected by flow cytometry and MTT method, and the damage and apoptosis of the two cell lines were observed. The results showed that the highly expressed FtMt had a protective effect on the cells against oxidative stress. Stenirri et al. (2012) found that there are mutant FtMt in some AMD patients. The conformation of mutant FtMt changes, which weakens the original antioxidant stress ability and causes oxidative damage to cells. Malondialdehyde level is a marker of oxidative stress level. Yildirim et al. (2004) detected the plasma malondialdehyde level in 30 patients with AMD and 60 healthy people. It was found that the plasma malondialdehyde level in patients with AMD is significantly higher than that in healthy people. The macular region of healthy people is rich in lutein, which can filter harmful light and reduce oxidative damage. This suggests that the occurrence of AMD is related to the decrease of lutein in macula. Gale et al. (2003) found that the pigment in the macular area of AMD patients decreased. It has also been reported that high-dose intake of lutein can help delay the loss of vision in patients with atrophic AMD, which proves that the development of AMD is related to the reduction of lutein (Yildirim et al., 2004).

### Immune inflammation

Recently, studies have shown that fundus inflammation leads to the production of vitreous membrane wart and the occurrence and development of AMD (Gale et al., 2003). Immunohistochemical study on vitreousmembrane wart showed that its components mainly include RPE cells, factors involved in complement pathway, acute phase molecules, main tissue soluble type II antigen complex, etc., (Wei et al., 2020). Some pathological components of atherosclerosis, Alzheimer’s disease and type 2 membranoproliferative glomerulonephritisare the same as thatofvitreous membrane warts, suggesting that the pathogenesis of these diseases may be similar to AMD. Some scholars believe that AMD may be a chronic persistent inflammatory disease. Local cell tissue damage leads to the damage of inner and outer retinal barriers and the release of inflammatory factors (Tavakoli et al., 2020). Wei et al. (2020) observed the vitreous membrane wart under the electron microscope and found that dendritic cells could be seen in the center of most vitreous membrane warts. Zhang et al. (2014) measured monocyte chemoattractant protein 1 and transforming growth factorβ1and interleukin-6 in venous blood of 55 patients with exudative AMD and 33 patients with age-related cataract by enzyme-linked immunosorbent assay (ELISA). The results showed that the levels of transforming growth factor β1 and interleukin-6 in venous blood of patients with exudative AMD are significantly higher than those of patients with age-related cataract. The level of interleukin-6 is significantly higher than that in age-related cataract patients. Studies have shown that macrophages are abundant in the choroid of AMD patients and isolated CNV tissues (ClearkinLRamasamy et al., 2019). ClearkinLRamasamy et al. (2019) found that in mice with reduced macrophages induced by laser, the expression of vascular endothelial growth factor (VEGF) is decreased due to the decrease of macrophages, which lead to the decrease of CNV production.

### Heredity and gene mutation

Ma et al. (2021) confirmed that heredity is related to the occurrence of AMDby studying on 840 elderly male twins. The early clinical manifestation of Sorsby macular dystrophy (SDF) is the thickening of Bruch’s membrane in the macular region and the deposition of lipid like substances. In the late stage, there are scars, neovascularization and hemorrhage in the macular region. SDF has many similarities with AMD and has become an important model to study the mechanism of AMD. As early as 1994, Weber et al. (1994) found that the gene causing AMDis located on chromosome 22q13.1 when studying a Canadian SDF family. Gene abnormalities in this region will lead to the disruption of the stability of extracellular matrix, the damage of retinal tissue and the generation of CNV. Studies have shown that a variety of complement factors in the complement system are related to the occurrence of AMD, including complement 3 (C3), complement factor H (CFH) and complement factor I (CFI) (Bhisitkul, 2006). Bhisitkul (2006) extracted DNA from venous blood of 119 patients with exudative AMD, 120 patients with early AMD and 140 healthy controls, and detected CFI gene by polymerase chain reaction (PCR) combined with restriction enzyme digestion analysis and DNA sequencing. The results showed that CFI is associated with the occurrence of AMD, and the CFI variant alleles in exudative AMD are less than those in the control group. Thus, CFI variant allele is a protective factor. Ithas been demonstratedby many studiesthatAMDis related to gene mutation, while the occurrence of AMD may also be related to the imbalance between inhibitory genes and regulatory genes (DorY and Keshet, 2001). The pathogenic gene of AMDisstill unclear, and further studies are needed to verify it.

### Hemodynamic changes

AMD patients, especially exudative AMD patients, have decreased choroidal blood flow and blood perfusion in the macular areaof the affected eye. RPE cells suffer from hypoxia and injurydue to insufficient perfusion, whichin turn leads to the formation of CNV and vascular exudation. Due to blood lipid deposition, arteriosclerosis and other reasons, it often leads to thickening of vascular wall, narrowing of vascular cavity and reduction of vascular wall compliance in the elderly, which leads to changes in hemodynamics and affects fundus blood perfusion. Sharma et al. (2021) used Doppler ultrasound to detect the hemodynamic changes of ophthalmic artery, central retinal artery and posterior ciliary artery ofthe affected eyes in 47 patients with AMD. It was found that theocular artery peak systolic velocity (PSV) of blood flowdecreased significantly, especially in patients with exudative AMD. With the aggravation of the disease, the ocular arterial resistance index (RI) also increased significantly. Abnormal perfusion of the choroid can lead to calcification and damage of Bruch’s membrane, accelerating lipid deposition, further impairing the function of RPE cells, and ultimately inducing AMD (ArjamaaONikinmaa et al., 2009). Cigarette smoke contains a large number of substances harmful to blood vessels, which is an important risk factor for inducing AMD. At the same time, smoking can promote the transformation of atrophic AMD into exudative AMD. Mei et al. (2009) analyzes the carotid hemodynamics of 50 active smokers, 44 passive smokers and 44 normal controls by vascular echo tracking (ET)technology. The results showed that the carotid pressure-strain elastic coefficient and stiffness of active smokers and passive smokers are higher than those of normal controls, while the carotid compliance of active smokers and passive smokers is significantly lower than that of normal controls.

## Vascular endothelial growth factor and age-related macular degeneration

The pathogenesis of AMD is not fully understood, and epidemiology shows that ethnicity, age, and smoking are definite risk factors, with a high prevalence in Caucasians and a low prevalence in Blacks, and no difference in prevalence between men and women (ArjamaaONikinmaa et al., 2009; Sharma et al., 2021). Numerous studies have shown that the VEGF/VEGFR pathway induces neovascularization and is an important pathogenesis of AMD. VEGF is a glycoprotein isolated from bovine pituitary follicular cells by Ferrara et al., in 1989, and seven kinds have been identified to date: VEGF (VEGF-A), VEGF-B to E, spinal cord-derived growth factor, and placental growth factor (PIGF). VEGF receptors include fms-like tyrosine kinase 1 (i.e., vascular endothelial growth factor receptor l, VEGFR1), fetal liver kinase insertion domain receptor (i.e., VEGFR2), VEGFR3, Neuropillin-1 (Npn-1) and Npn-2 (Mei et al., 2009). VEGF promotes neovascularization mainly by binding to ligand families which containing one or both VEGFR1 and VEGFR2.

VEGF-A is the strongest pro-angiogenic growth factor known at present. Its role is mainly to promote angiogenesis, proliferation and survival, and increase microvascular permeability, while VEGFR2 is the main mediator of angiogenesis (Mei et al., 2009). PIGF can selectively bind to VEGFR1 and be abundantly expressed in endothelial cells, thus accelerating VEGF-A-induced endothelial cell proliferation. In addition to directly mediating angiogenesis, VEGF can also stimulate the expression of matrix metalloproteinases in vascular endothelial cells, help neovascularization invade tissues by degrading extracellular matrix, make CNV grow continuously, and promote the chemotaxis of monocytes and macrophages (Wang et al., 2013a; Mohan et al., 2013).

In the early stage of exudative AMD, theincreased VEGF expression can be detected in both the RPE and the outer nuclear layer of the macula, and elevated VEGF levels are also seen in surgically resected CNV lesions by immunohistochemical methods, all of which suggest an important role for VEGF in exudative AMD. With the development of AMD, choroidal microcirculation is often impaired, leading to tissue ischemia and hypoxia. Shibuya and claesson-welsh (2006) showed that VEGF mRNA transcript levels are upregulated under the condition of retinal hypoxia, and correspondingly the expressionsof VEGFR1 and VEGFR2 are increased. In the normal physiological state, VEGFR1 inhibits the over-phosphorylated expression of VEGFR2, whereas it exhibits a promoting effect in the pathological state and increases vascular permeability. The two interact to promote neovascularization.

## Anti-vascular endothelial growth factor drugs for age-related macular degeneration

At present, anti-VEGF drugs used in clinic and R&D are roughly divided into drugs that block the binding of VEGF and VEGFR (such as anti-VEGF aptamer and anti-VEGF monoclonal antibody or antibody fragment), drugs that block important kinases downstream of VEGF pathway signal transduction and drugs that inhibit VEGF gene expression (Peng, 2021; ZarbinMAHill et al., 2021). By blocking the activation of VEGF downstream pathway, anti-VEGF drugs can reduce the increase of vascular permeability caused by the increase of VEGF, inhibit the growth of CNV, alleviate macular edema and improve the visual acuity of patients with exudative AMD. However, a number of clinical trials of several anti-VEGF drugs currently used in clinic show that 1/4 of patients still have no response to treatment, and even their vision continues to decline after treatment, eventually becoming blind (Mettu et al., 2020b). Meanwhile, anti-VEGF drugs often need to be injected repeatedly on a monthly basis, which increases the risk of complications.

At present, there is no specific treatment for AMD, especially for atrophic AMD. Most treatments are for CNV associated with exudative AMD. The formation mechanism of CNV is not very clear. The current research have showed that inflammation plays an important role in the occurrence and development of AMD (ArjunanPSwaminathan et al., 2021). Inflammatory reaction promotes the over-regulation of cells and a variety of growth factors, leading to pathological vascular proliferation and CNV. Among the growth factors, VEGF is the most powerful promoter of ocular neovascularization (ZhuLParker et al., 2020). The continuous research on the mechanism of CNV for AMD and the R&D of anti-VEGF drugs, that is, applying anti-VEGF drugs to prevent choroidal neovascularization in the treatment of wet or neovascularAMD (Wang et al., 2013b), shows that the level of VEGF in aqueous humor of AMD patients is significantly higher than that of normal people. VEGF is an important factor to promote the formation of CNV. Inhibiting VEGF can effectively inhibit the formation of CNV and reduce vascular exudation. Anti-VEGF drugs are the main method to treat exudative AMD. At present, anti-VEGF drugs mainly include the following:

### Avastin

Avastin, a recombinant human VEGF monoclonal antibody, was approved by the U.S. Food and Drug Administration (FDA) in February 2004 and is the first drug approved for marketing in the United States to inhibit tumor angiogenesis. Because of its structural similarity to Lucentis, it has been applied by some clinicians to ophthalmic neovascular diseases and has shown good safety and efficacy. Hong et al. (2020) showed that intravitreal injection of Avastin in 22 patients (22 eyes) is safe and effective in treating exudative AMD, significantly reducing macular edema and improving visual acuity at a 6-months follow-up.

### Lucentis

Lucentis is the second generation of recombinant human VEGF monoclonal antibody, which can specifically bind VEGF-A and has higher affinity than Avastin. As one of the anti-VEGF drugs, clinical studies have shown that Lucentis can effectively reduce neovascular exudation, reduce macular edema, and improve or maintain the existing visual acuity (Hong and Liu, 2010). Chu et al. (2015) analyzed the clinical data of 46 patients (47 eyes) with exudative AMD who received intravitreal injection of Lucentis and the best corrected visual acuity are <0.05. It was found that after treatment, 61.7% (29/47) of the patients had improved visual acuity, 31.9% (15/47) of the patients had stable visual acuity, and the retinal thickness in the macular center decreased significantly compared with that before treatment.

### Aflibercept

Aflibercept, marketed in 2011, is a recombinant VEGF receptor fusion protein consisting of Domain2 of VEGF-R1 and Domain3 of VEGF-R2 and the Fc segment of IgG1, which can specifically bind VEGF-A, VEGF-B, and platelet-derived growth factor (PDGF). Some studies have shown that treatment with Aflibercept in patients with exudative AMD, who were poorly treated with other anti-VEGF drugs, can improve visual acuity and reduce macular edema in the short term (Singh et al., 2014). Heier et al. (2012) treated 2419 subjects with intravitreal injection of Lucentis and Aflibercept, and compared their efficacy and safety. The results showed that Aflibercept and Lucentis have similar efficacy and safety.

### Conbercept

Conbercept is the first independently developed anti-VEGF drug in China, a novel receptor fusion protein, which was approved by ChinaNational Medical Products Administration in 2013 for the treatment of exudative AMD. Conbercept specifically binds VEGF-A, VEGF-B, VEGF-C and human placental factor (PIGF). Zhang et al. (2008) applied Biacore and ELISA assays to compare the affinity of Conbercept and Avastin for VEGF-A and PIGF. It was found that Conbercept had higher affinity for VEGF-A than Avastin, and Avastin did not have affinity for PIGF, suggesting that Conbercept has better efficacy and safety in the treatment of exudative AMD. Yu et al. (2015) treated 20 patients with exudative AMD with intravitreal injection of Conbercept once/month for 3 consecutive injections, and after 6 months of follow-up, the patients had significantly improved visual acuity and reduced macular central recess thickness, and no serious adverse effects were observed.

### Pegaptanib

Pegaptanib, the first anti-VEGF drug used in ophthalmology, is an RNA aptamer consisting of 28 bases which specifically binds VEGF-A165. Gragoudas et al. (2004) found that Pegaptanib stabilized but did not improve visual acuity in patients with exudative AMD.

### Other drugs

Vatalanib (PTK787) is a potent inhibitor of all known VEGF receptor tyrosine kinases, including VEGFR1, VEGFR2 and VEGFR3. The bioavailability of Vatalanib is high. Preclinical trials and phase I clinical trials showed that Vatalanib has inhibitory effect on CNV and good safety. At present, a trial combined Vatalanibwith PDT is under way. Regorafanib mainly targets VEGF1/3, PDGF-B and fibroblast growth factor receptor 1. The eye drops made from this drug have proved their safety in phase I clinical trials and are now in phase II clinical trials (Hong et al., 2020). As a small molecule preparation, RTKIhas the advantages of easy absorption, simple synthesis and suitable for long-term medication. However, due to its inhibitory effect on a variety of kinases in human body, its specificity is poor and may cause serious adverse reactions. Its clinical efficacy still needs to be further studied. With the approval of anti-VEGF drugs for ophthalmology, AMD patients have ushered in the dawn of hope (Schopf et al., 2013). In order to improve the long-term prognosis and quality of life of patients to the greatest extent, reducing the number of intraocular injections, prolonging the treatment interval, enhancing the efficacy and simplifying the mode of administration are still the hot and difficult areas of concern in the treatment of wet AMD. Although new drugs have been developed to improve at different levels, better treatment schemes still need to be explored in the future.

## Prospect

The occurrence and development of AMD is an extremely complex process, in which a large number of regulatory factors and cytokines are involved. Most of the existing treatments are for its concomitant CNV (Yla et al., 2021). Especially, the anti-VEGF drugs have shown good efficacy and application prospect, and have becomea recognized first-line clinical treatment drug (Kc et al., 2020). However, the commonly used anti-VEGF drugs need frequent intravitreal injection, which may be accompanied by serious adverse reactions, thus bringing huge economic burden to patients. In order to improve these problems, researchers have made major breakthroughs in exploring new anti-neovascularization targets, multi-path combination drugs and new administration methods, such as Lucentisextended-release device, combinationwith PDGF target inhibitors, and various drugs developed, such as oral preparation X-82, small molecule preparation brolucizumab, apiciparpegol and eye drops made of VEGF receptor tyrosinase inhibitor pazopanib, have successively entered phase IIand Phase III clinical trials (Waters et al., 2021). However, just as the drug extended-release devices need to overcome the compatibility with tissue and pharmacological and toxicological reactions, and the phase III clinical trial of fovista combined with Lucentis and the phase II clinical trial of pazopanib ended in failure (Wu et al., 2019; Mettu et al., 2020c). The pharmacodynamics, pharmacokinetics and safety of these emerging drugs or administration methods need to be further studied. With the further understanding of the pathogenesis of AMD and the development of genetic technology and bioscience, it is expected that more effective, safer, more lasting and simplified drugs or treatments will be used in clinic as soon as possible and the combination of multiple methods is the main trend in the treatment of AMD in the future.
